# Hyperdiverse Gene Cluster in Snail Host Conveys Resistance to Human Schistosome Parasites

**DOI:** 10.1371/journal.pgen.1005067

**Published:** 2015-03-16

**Authors:** Jacob A. Tennessen, André Théron, Melanie Marine, Jan-Ying Yeh, Anne Rognon, Michael S. Blouin

**Affiliations:** 1 Department of Integrative Biology, Oregon State University, Corvallis, Oregon, United States of America; 2 CNRS, UMR 5244, Ecologie et Evolution des Interactions (2EI), Université de Perpignan Via Domitia, Perpignan, France; Cornell University, UNITED STATES

## Abstract

Schistosomiasis, a neglected global pandemic, may be curtailed by blocking transmission of the parasite via its intermediate hosts, aquatic snails. Elucidating the genetic basis of snail-schistosome interaction is a key to this strategy. Here we map a natural parasite-resistance polymorphism from a Caribbean population of the snail *Biomphalaria glabrata*. In independent experimental evolution lines, RAD genotyping shows that the same genomic region responds to selection for resistance to the parasite *Schistosoma mansoni*. A dominant allele in this region conveys an 8-fold decrease in the odds of infection. Fine-mapping and RNA-Seq characterization reveal a <1Mb region, the Guadeloupe Resistance Complex (GRC), with 15 coding genes. Seven genes are single-pass transmembrane proteins with putative immunological roles, most of which show strikingly high nonsynonymous divergence (5-10%) among alleles. High linkage disequilibrium among three intermediate-frequency (>25%) haplotypes across the GRC, a significantly non-neutral pattern, suggests that balancing selection maintains diversity at the GRC. Thus, the GRC resembles immune gene complexes seen in other taxa and is likely involved in parasite recognition. The GRC is a potential target for controlling transmission of schistosomiasis, including via genetic manipulation of snails.

## Author Summary

Schistosomes are water-borne blood-flukes that are transmitted by snail vectors. They infect over 200 million people in more than 70 countries and cause severe and chronic disability. Snails naturally vary in resistance to this parasite even within species, so bolstering snail resistance in the wild would block transmission. We artificially selected snails for resistance and observed a rapid evolutionary response, with the greatest change occurring in the same genomic region in two independent trials. We subsequently confirmed that the selected haplotype conveys resistance to infection by schistosomes. The extraordinarily high sequence divergence among haplotypes in this region appears to be elevated due to ongoing natural selection, likely via host-parasite co-evolution. We observed the highest variation in genes encoding putative parasite recognition proteins, suggesting that these control the resistance phenotype in a manner reminiscent of immune gene complexes in other taxa. Thus, this gene cluster presents a potential new target to interfere with parasite transmission at the vector stage.

## Introduction

Schistosomiasis is by far the most important helminth parasitic disease of humans. Schistosomes infect over 200 million people worldwide [1,2], causing a chronic, debilitating disease that can lead to lifelong disability [3,4]. The disability-adjusted-life years lost to this disease are estimated at 13–56 million, a value rivaling that of malaria [3]. There are no effective vaccines against schistosomes, and effective treatment still relies on regular dosing with a single drug, praziquantel [5]. Praziquantel resistance in schistosomes can be easily selected for in the lab, suggesting that natural populations which infect humans could also evolve drug resistance [6]. In fact, there is now credible evidence of reduced drug susceptibility in some heavily-treated human populations [7]. This problem may increase substantially as mass treatment with praziquantel escalates under initiatives such as the Schistosomiasis Control Initiative [8] and the Gates Foundation’s SCORE project (http://score.uga.edu). Alternate control strategies are therefore needed, including tactics for blocking transmission via the aquatic snails that serve as intermediate hosts.

Understanding the molecular mechanisms by which snails and schistosomes interact will be the key for new approaches to interrupt transmission [9]. Most molecular research to date has focused on the schistosome parasite *Schistosoma mansoni* and its New World snail host *Biomphalaria glabrata*, recently aided by the newly sequenced genomes of both species ([10]; https://www.vectorbase.org/organisms/biomphalaria-glabrata) and transcriptomic studies [11,12]. Several lines of evidence demonstrate that resistance to infection is highly heritable in snails [13–17]. First, infection rates in inbred snail lines are consistent and typically either 0% or close to 100% [13,14]. Second, artificial selection experiments can produce snails with significantly increased or decreased susceptibility in a few generations [15,17]. Third, resistance to infection can be mapped to genetic markers in linkage crosses [16]. However, despite recent advances [18–20], our knowledge of *B*. *glabrata*-*S*. *mansoni* interactions lags behind that of other host-parasite systems such as mosquito-Plasmodium [21,22]. Expression levels of some genes are known to influence resistance of *B*. *glabrata* to *S*. *mansoni* (FREPs, [23,24]; Hsp 90, [25]). However, to date there is only one genic locus known at which allelic variation associates with resistance (*sod1*, [26,27]), and the causality of this association still needs to be proven. Furthermore, there is substantial *B*. *glabrata*-strain by *S*. *mansoni*-strain (G × G) interaction in compatibility (i.e. one strain of *B*. *glabrata* can be highly resistant to one strain of *S*. *mansoni*, but highly susceptible to another, and vice versa) [28–32]. This pattern may reflect different per-strain combinations of highly diverse coevolving loci [33] which could conform to one of several models of host-parasite genotype matching [34].

In lab populations of *B*. *glabrata* and *S*. *mansoni* from natural populations in Guadeloupe, West Indies, only 40–50% of snails can be infected, no matter how many parasite miracidia are used to challenge them [33]. This scenario is consistent with a simple model of host-parasite genotype matching, in which the parasite population lacks the ability to be compatible with certain alleles in the host population [33]. Here we identify and characterize this resistance polymorphism in the snail population at the molecular level.

## Results

### Selection for resistance produces rapid phenotypic change

**Fig 1:**
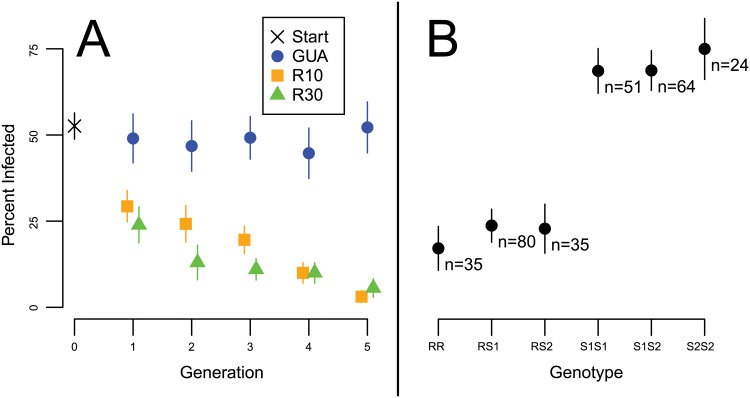
Resistance phenotypes. Standard errors of proportions are indicated by vertical bars. (A) Susceptibility declined rapidly over five generations in snail lines exposed to 10 (R10) or 30 (R30) miracidia, when only uninfected snails were allowed to contribute to the next generation (N = 46–100). A control line (GUA) that was not exposed to the parasite showed no comparable change (N = 46–65). (B) Among 289 snails, the R allele at the GRC locus *grc1* is strongly correlated with resistance in a dominant fashion. There are no significant differences in resistance among genotypes with R, nor among genotypes without R.

We started with a laboratory population of snails approximately ten generations removed from the wild, which had originated from hundreds of wild Guadeloupe snails and had been maintained as a randomly-mating population in the hundreds at the Université de Perpignan (“Guadeloupe laboratory population”) [33]. We selected two independent lines for resistance by challenging individual snails with either 10 (line R10) or 30 (line R30) miracidia and allowing only uninfected snails to found the next generation (S1 Table). Susceptibility (% of snails infected) dropped from approximately 50% (53% +/- 4) to under 10% (3% +/- 2 for R10; 6% +/- 3 for R30) over five generations in the selection lines, but remained at approximately 50% (52% +/- 7) in the unselected control line GUA (Fig. 1A). Linear regression of generation time on susceptibility (logit transformed) showed no significant effect for GUA (p > 0.1), but a significantly negative slope for both R10 (odds of infection each generation decrease by 45% (95% CI = 36–54%); p < 0.01) and R30 (odds of infection each generation decrease by 42% (95% CI = 28–53%); p < 0.01). A combined regression with both selection lines showed no significant effect of population (R10 vs. R30) on susceptibility (p > 0.1).

### The same genomic region responds repeatedly to selection

**Fig 2:**
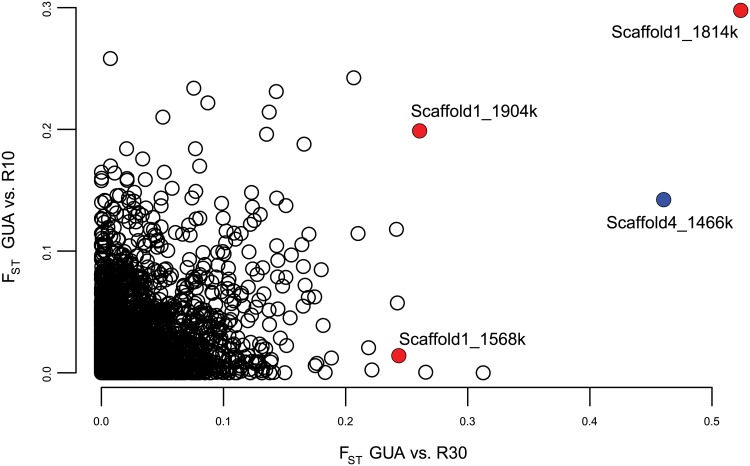
F_ST_ values between the unselected control line (GUA) and each selected line (R10 and R30). Three null RAD markers, aligned with BWA [65] to Scaffold1, all showed unusually high F_ST_ in one or both comparisons (red). These markers conform to perfect linkage disequilibrium with the haplotypes as determined by Sanger sequencing, with the different F_ST_ values owing only to the error inherent in estimating allele frequencies from null markers. An additional unaligned marker with high F_ST_ in both comparisons was identified with Stacks [35] and found to reside on Scaffold4 (blue).

In samples of 28 individuals each from GUA, R10 and R30, we observed 6573 informative RAD markers that aligned to the reference genome, including 4142 with codominant (two observable alleles) variants (SNPs or small indels) and 2431 null markers. The same null marker, aligning to Scaffold1, Site 1.814Mb, showed the highest allele frequency difference for both the R30-GUA comparison (F_ST_ = 0.52) and the R10-GUA comparison (F_ST_ = 0.30), with the allele changing frequency in the same direction for both selection lines (Fig. 2). In 100 bootstrap replicates in which 28 samples of each population were chosen randomly with replacement, this marker was the top F_ST_ outlier for the R30-GUA comparison 84% of the time (95% CI of F_ST_ = 0.38–0.67), and for the R10-GUA comparison 8% of the time (95% CI of F_ST_ = 0.15–0.50). We selected this top outlier for further study. We next looked at the additional markers on Scaffold 1 and found two other null markers <250kb away showing relatively high F_ST_ (Fig. 2). We supplemented our analysis with Stacks [35], a program that aligns reads to each other rather than to a reference genome, in order to find divergent markers on reads with low similarity to the reference genome. Because the genomic sequence flanking Stacks markers is unknown, they can be difficult to confirm with PCR, so we did not employ Stacks as a stand-alone analysis. However, Stacks revealed an additional RAD marker showing high allele frequency difference (R30-GUA: F_ST_ = 0.46; R10-GUA: F_ST_ = 0.14), and high linkage disequilibrium (LD) to Scaffold1, Site 1.814Mb (r = 0.94, p < 10^-15^), which had been too divergent to align unambiguously to the reference genome but which showed sequence similarity to Scaffold4, Site 1.466Mb (Fig. 2).

### This genomic region is strongly associated with resistance

**Fig 3:**
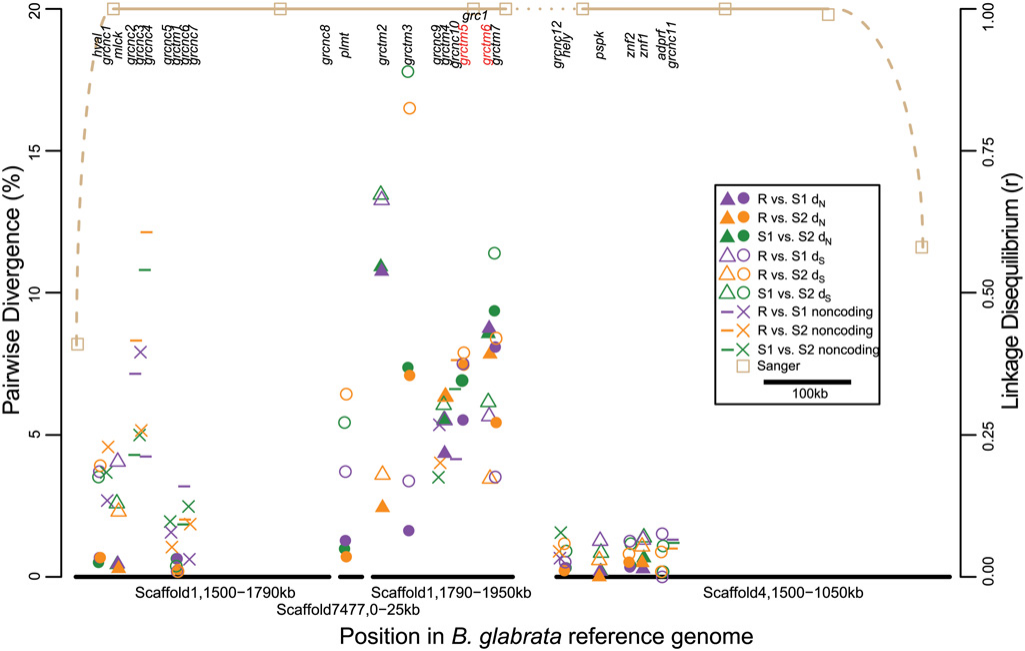
Pairwise divergence of GRC genes. Genes are aligned to their approximate genomic position on the x-axis, staggered slightly for ease of visualization. Scaffold7477 has been inserted into its inferred position within Scaffold1. Scaffold4 has been inverted relative to its arbitrarily designated reference genome orientation to indicate that only the end of this scaffold is part of the GRC. Pairwise divergence for all three haplotype combinations (indicated by color) is shown in the left y-axis. Gene symbols vary for ease of distinguishing adjacent genes, and to show divergence type: solid symbols represent nonsynonymous divergence (d_N_) in coding genes, open symbols represent silent (synonymous and noncoding) divergence in coding genes (d_S_), and other symbols represent divergence in noncoding genes. Only the six TM1 genes in the center of the region of association (*grctm2*–7) show high (>1%) d_N_. The names of the two most promising candidates, *grctm5* and *grctm6*, are highlighted in red. Brown lines and squares indicate the boundaries of the GRC region of statistical association as determined by Sanger sequencing markers: for these markers, the right y-axis indicates linkage disequilibrium correlation (r; Table 1) with marker locus *grc1* (labeled). The gap between Scaffold1 and Scaffold4 (dotted line) is of unknown size, but contains no expressed genes with sequence or expression differences among haplotypes, as these would have been detected in our RNA-Seq analysis.

We determined the susceptibility/resistance phenotype of 289 additional snails from the Guadeloupe laboratory population by challenging each with 20 miracidia: 159 were resistant (uninfected) and 130 were susceptible (infected). We genotyped them at one of the high-F_ST_ RAD markers on Scaffold1 (“*grc1*”) using PCR and Sanger sequencing. All Sanger genotypes could be unambiguously phased by eye and were found to comprise three alleles (multi-SNP haplotypes), all of intermediate frequency. Allele R (“resistance”) with a frequency of 32.0% (+/- 3.4%), showed a strong negative correlation with infection (Fig. 1B) and was dominant to the two S (“susceptibility”) alleles, S1 (42.6% +/- 3.2% frequency) and S2 (25.4% +/- 3.6% frequency). Specifically, snails without an R allele (n = 139) had a 70 (± 4) % chance of infection, while snails with at least one R allele (n = 150) had a 22 (± 3) % chance of infection (r^2^ = 0.23; odds ratio = 8.2; Fisher’s exact test, p < 10^-15^). There was no significant difference in infection between RR homozygotes and RS heterozygotes (Fisher’s exact test, p > 0.1). Similarly, Sanger sequencing and genotype phasing by eye revealed three alleles (multi-SNP haplotypes) in a PCR-amplified region of Scaffold4 (“*grc2”*), one of which was in near-perfect LD with allele R: only one individual out of 289 showed recombination between Scaffolds 1 and 4 (r = 0.996, p < 10^-15^). The remaining two *grc2* alleles were in modest LD with S1 and S2 (r = 0.572 among the 139 SS snails, p < 10^-12^). We genotyped a subset of 94 snails at additional sites on Scaffold1 and Scaffold4 (Table 1; Fig. 3), and found alleles in perfect LD with allele R extending from sites 1.541–1.940Mb on Scaffold1 and sites 1.187–1.468Mb on Scaffold4, with the exception of a single recombinant at Scaffold4_1.187Mb. LD declined rapidly further upstream on both scaffolds, beginning at site 1.501Mb on Scaffold1 and at site 1.081Mb on Scaffold4. The different F_ST_ values among the RAD markers on these scaffolds (Fig. 2) is consistent with the imprecision of genotyping with null RAD tags (e.g. null heterozygotes cannot be identified), rather than different allele frequencies in the sample. Thus, the ends of these two scaffolds are tightly linked and probably either adjacent or nearly so, forming a region of > 0.7Mb in near-perfect LD and showing a strong association with resistance. We refer to this region as the Guadeloupe Resistance Complex (GRC).

**Table 1:** Markers used in Sanger sequencing to delimit borders of GRC.

Site^a^	r^b^
Scaffold1_1501k	0.41
Scaffold1_1541k	1
Scaffold1_1732k	1
Scaffold1_1904k^c^	1
Scaffold1_1940k	1
Scaffold4_1468k	1
Scaffold4_1305k^d^	1
Scaffold4_1187k	0.99
Scaffold4_1081k	0.58

^a^Position in *B*. *glabrata* reference genome v. BglaB1 (scaffold, followed by site position in kb)

^b^correlation coefficient (r) with *grc1* among 94 snails

^c^ *grc1*

^d^ *grc2*

### GRC genes encode proteins with likely immunological roles

We performed RNA-Seq on outbred, parasite-unchallenged individuals of each genotype from an Oregon State University population derived from the original Guadeloupe laboratory population. The purpose of the RNA-Seq analysis was to facilitate gene annotation of the GRC, to obtain coding sequence for each allele, and to identify genes that might be differentially expressed between haplotypes. We sequenced 18 RR homozygotes, 9 S1S1 homozygotes, and 9 S2S2 homozygotes, all showing perfect LD among all three alleles at *grc1* and *grc2*. In order to exhaustively characterize differences in either sequence or expression among haplotypes, we identified 31bp sequences (31-mers; the largest size possible in our analysis pipeline) showing significantly higher counts between RR and SS (8639 31-mers), between S1S1 and RR (5586 31-mers), and between S2S2 and RR (14976 31-mers). The large excess of 31-mers in S2S2 vs. RR, relative to the other comparisons, stems from the fact that LD between these two haplotypes extends ~0.7Mb farther upstream on Scaffold4. We ignored these 31-mers that align upstream of Scaffold4_1.1Mb because recombination between R and S1 indicates that they do not contain the causal variant. We assembled transcripts from 27 genes (defined as a transcribed sequence >500bp), including 15 coding and 12 putative noncoding (no open reading frame >500bp; designated *grcnc* for “Guadeloupe resistance complex noncoding”) genes that encompassed most of these 31-mers (95% for R, 90% for S1, and 31% for S2; Table 2). Nearly all transcripts aligned to the GRC region on Scaffold1 and Scaffold4, with two exceptions: one transcript (*plmt*) aligned to Scaffold7477, and another (*grctm2*) aligned partially to Scaffold1 and partially to Scaffold7477. However, SNPs in these transcripts were in perfect LD with the GRC, indicating that this section of Scaffold7477 (at least 6kb in size) occurs within the GRC.

**Table 2:** Genes in the GRC.

Gene^a^	Description^b^	E-value^c^	Site^d^	ORF^e^	RS1 d_N_ ^f^	RS1 d_S_ ^g^	RS2 d_N_ ^h^	RS2 d_S_ ^i^	S1S2 d_N_ ^j^	S1S2 d_S_ ^k^
*hyal*	hyaluronidase	1.00E-162	Scaffold1 1520–1531k	1479	0.68	3.71	0.68	3.92	0.51	3.52
*grcnc1*	noncoding	NA	Scaffold1 1534–1536k	0	NA	2.69	NA	4.58	NA	3.68
*mlck*	myosin light chain kinase	2.00E-12	Scaffold1 1541–1553k	5604	0.46	4.06	0.29	2.3	0.43	2.59
*grcnc2*	noncoding	NA	Scaffold1 1571–1573k	0	NA	7.07	NA	8.24	NA	4.21
*grcnc3*	noncoding	NA	Scaffold1 1573–1574k	0	NA	7.92	NA	5.16	NA	5
*grcnc4*	noncoding	NA	Scaffold1 1574–1575k	0	NA	4.15	NA	12.04	NA	10.71
*grcnc5*	noncoding	NA	Scaffold1 1607–1609k	0	NA	1.57	NA	1.05	NA	1.95
*grctm1*	single-pass transmembrane	NA	Scaffold1 1610–1621k	1002	0.64	0.19	0.26	0.19	0.64	0.38
*gcrnc6*	noncoding	NA	Scaffold1 1624–1626k	0	NA	3.1	NA	1.93	NA	1.76
*grcnc7*	noncoding	NA	Scaffold1 1626–1627k	0	NA	0.62	NA	1.86	NA	2.48
*grcnc8*	noncoding	NA	Scaffold1 1786–1787k	0	NA	NA	NA	NA	NA	NA
*grctm2*	receptor-type tyrosine-protein phosphatase	1.00E-28	Scaffold1 1792–1806k, Scaffold7477 2–3k	2013	10.75	13.26	2.44	3.6	10.91	13.45
*grctm3*	chitinase	6.00E-12	Scaffold1 1821–1837k	864	1.63	3.38	7.1	16.5	7.38	17.79
*grcnc9*	noncoding	NA	Scaffold1 1865–1867k	0	NA	5.36	NA	4.02	NA	3.51
*grctm4*	chitinase	5.00E-13	Scaffold1 1863–1877k	1104	4.35	5.52	6.37	6.35	5.55	6.06
*grcnc10*	noncoding	NA	Scaffold1 1884–1885k	0	NA	4.06	NA	7.55	NA	6.53
*grctm5*	chitinase	9.00E-14	Scaffold1 1886–1895k	855	5.53	7.51	7.46	7.9	6.94	6.92
*grctm6*	single-pass transmembrane	NA	Scaffold1 1906–1936k	2013	8.74	5.66	7.85	3.46	8.57	6.16
*grctm7*	single-pass transmembrane	NA	Scaffold1 1922–1934k	1359	8.1	3.52	5.44	8.4	9.36	11.39
*grcnc12*	noncoding	NA	Scaffold4 1497–1500k	0	NA	0.66	NA	0.9	NA	1.56
*hely*	hemolysin	3.00E-16	Scaffold4 1477–1501k	2385	0.32	0.52	0.22	1.17	0.32	0.91
*pspk*	paraspeckle component	5.00E-89	Scaffold4 1441–1456k	2028	0.19	1.27	0	0.59	0.19	0.85
*znf2*	zinc finger	0	Scaffold4 1406–1423k	5400	0.35	1.26	0.52	0.81	0.4	1.17
*znf1*	zinc finger	1.00E-130	Scaffold4 1395–1403k	2256	0.28	1.31	0.5	1.07	0.67	1.39
*adprf*	ADP ribosylation factor GTPase-activating protein	5.00E-151	Scaffold4 1368–1387k	1416	0	1.52	0.18	0.88	0.18	1.09
*grcnc11*	noncoding	NA	Scaffold4 1365–1366k	0	NA	1.22	NA	0.91	NA	1.11
*plmt*	palmitoyltransferase	4.00E-68	Scaffold7477 5–8k	903	1.28	3.71	0.71	6.44	0.99	5.44

^a^gene name following Bayne [70]

^b^for coding genes, putative protein description based on BLAST hits and secondary structure

^c^lowest E-value for protein sequence BLASTed against NCBI non-redundant protein sequences. "NA" means non-coding or no E-values < 10^-2^

^d^approximate location in *B*. *glabrata* reference genome v. BglaB1 via BLAST (site position in kb)

^e^size of open reading frame in bp

^f^nonsynonymous divergence between R and S1 (%)

^g^silent (synonymous and noncoding) divergence between R and S1 (%)

^h^nonsynonymous divergence between R and S2 (%)

^i^silent (synonymous and noncoding) divergence between R and S2 (%)

^j^nonsynonymous divergence between S1 and S2 (%)

^k^silent (synonymous and noncoding) divergence between S1 and S2 (%)

**Fig 4:**
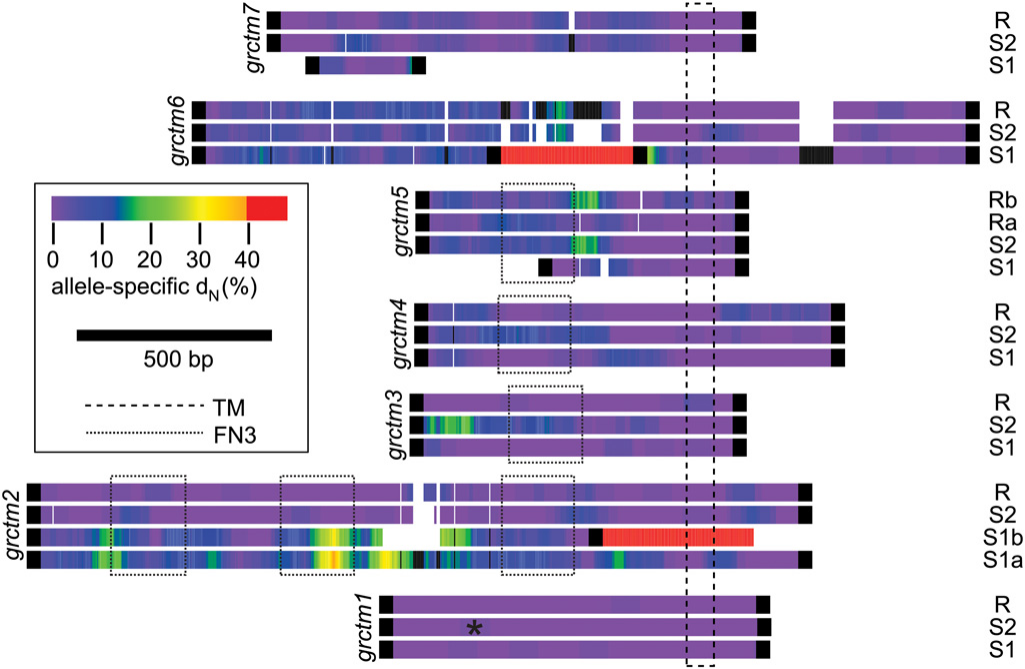
Allele-specific nonsynonymous substitution among alleles of the seven TM1 genes. Alleles of the same gene are aligned to each other (gaps indicated by whitespace), but across genes only the transmembrane domain (TM) is aligned (extracellular (N-terminal) regions are to the left of the TM). If a haplotype includes two copies of a gene that is single-copy on the other haplotypes, both copies are shown (e.g. gene *grctm5* is duplicated on the R haplotype, so we label those sequences Ra and Rb). For each allele, we calculated allele-specific nonsynonymous substitution (i.e. divergence from the inferred ancestral sequence) in 75bp sliding windows, indicated by color (“allele-specific d_N_”). Regions with no sequence similarity are indicated in red. Substitution across a 75bp window could not be calculated in black sections. Fibronectin III domains (FN3) are shown. The premature stop codon in *grctm1* S2 is shown with an asterisk. Nonsynonymous substitution is extremely high across the TM1 genes, exceeding 15% in some windows for all genes except *grctm1*, and occasionally reaching over 30%. Only *grctm5* and *grctm6* show high nonsynonymous substitution specific to R alleles.

With one exception (described below), all genes appeared in all three genotypes, although in some cases the orthologous transcript was under 500bp due to apparent truncation, and in some cases multiple isoforms of the same transcript were observed for some genotypes but not others (S1 Fig). The coding genes represented a diversity of protein families, including hyaluronidase, myosin light chain kinase, hemolysin, paraspeckle component, zinc finger, ADP ribosylation factor GTPase-activating protein, and palmitoyltransferase (Table 2). Other coding genes showed low or no similarity to any characterized sequence. Seven genes, all on Scaffold1 between positions 1.6–2.0Mb (middle of the GRC), show similar secondary structure, all encoding single-pass transmembrane proteins (TM1s) with >50 residues on either side of the transmembrane domain, and with a large (>50% of the protein) N-terminal (extracellular) domain in which β-strand residues are prevalent (>25% of residues and >1.5 times as common as α-helix residues). TM1 proteins are a structural class seen in a wide variety of protein families with roles in processes like cell migration, adhesion, and growth, and typically acting as receptors for extracellular signals [36]. Notably, TM1s play an important role in the immune system, and include B- and T-cell receptors, Fc receptors (binding the fragment crystallizable region of antibodies), some major histocompatibility complex (MHC) receptors, and Toll-like receptors, all of which are membrane-bound receptors recognizing foreign molecules [36]. Four of the TM1 genes in the GRC (*grctm2*, *grctm3*, *grctm4*, and *grctm5*) encode fibronectin III domains (DELTA-BLAST, E < 10^-3^; Fig. 4). Although the TM1 genes in the GRC do not all share primary sequence similarity, we designated them *grctm1-grctm7* for “Guadeloupe resistance complex transmembrane” due to their similar secondary structure.

### Low expression variation among GRC alleles

In the parasite-unchallenged snails, although expression varied substantially among genes (per-sample, per-site depth from 1x to >50x), expression differences among the three genotypes were low (S1 Fig, S2 Fig). We performed 90 t-tests for expression depth among genotypes in the unchallenged snails, and thus employed a Bonferroni-corrected α value of 0.0006. There was only a single instance of a gene not observed in all genotypes, and it was a short (605bp), noncoding sequence, *grcnc8*, observed only in RR snails at relatively low coverage (depth = 1.1x) (t-test between RR and SS, p<10^-15^). Only one other gene, the non-coding *grcnc9*, showed a >2-fold difference between RR (4.9x) and both SS (0.8x, 0.9x) genotypes (t-test, p<10^-9^). To be conservative with respect to identifying candidate genes, we also noted genes with expression differences that would be significant if uncorrected for multiple tests. There were two such genes showing small (<2 fold) differences between RR and both SS genotypes in the same direction: the non-coding *grcnc5* (RR = 9.7x, S1S1 = 6.6x, S2S2 = 5.0x; p<10^-2^), and the TM1 gene *grctm6* (RR = 1.2x, S1S1 = 2.2x, S2S2 = 2.4x; p<10^-2^). A few other genes showed >2-fold expression differences in some genotype comparisons, but not in both RR-S1S1 and RR-S2S2 comparisons.

In order to test whether any expression differences are only apparent during parasite challenge, we also performed RNA-Seq on pooled DNA from six families (3 RR, 1 S1S1, 2 S2S2) at 2 and 6 hour intervals after exposure to miracidia. We performed 62 t-tests for expression depth among genotypes in these challenged snails, and thus employed a Bonferroni-corrected α value of 0.0008. Expression differences among haplotypes appeared slightly greater in challenged snails than in unchallenged snails, but still low overall and not significant. As with unchallenged snails, we conservatively noted all genes with expression differences that would be significant if uncorrected for multiple tests. There were 4 genes with greater expression in SS families: *grcnc2* at both intervals (2 hr RR = 2.1x, 2 hr SS = 3.6x, 6 hr RR = 1.6x, 6 hr SS = 4.3x; p < 0.05 for both), *grctm6* at 6 hr (RR = 1.6x, SS = 4.5x; p < 0.05); *adprf* at 6 hr (RR = 13.6x, SS = 17.2x; p < 0.05) and *hely* at both intervals (2 hr RR = 1.9x, 2 hr SS = 4.2x, 6 hr RR = 1.6x, 6 hr SS = 3.0x; p < 0.05 for both). Only *grcnc8*, which was absent in both challenged and unchallenged SS snails, was higher in challenged RR families (2 hr RR = 0.8x, 6 hr RR = 0.9x). Additionally, there were 5 genes with >2-fold but nonsignificant (even if uncorrected) challenged expression differences among RR and SS snails: *hyal* at both intervals (2 hr RR = 2.8x, 2 hr SS = 9.9x, 6 hr RR = 3.3x, 6 hr SS = 8.5x), *grcnc4* at 2 hr (RR = 7.9x, SS = 3.7x), *grcnc6* at 2 hr (RR = 12.3x; SS = 3.5x), *grcnc9* at both intervals (2 hr RR = 1.2x, 2 hr SS = 0.4x, 6 hr RR = 1.0x, 6 hr SS = 0.4x), and *grctm6* at 2 hr (RR = 1.8x, SS = 3.8x). Notably, there were no coding genes with increased expression in RR families.

### High sequence variation among GRC alleles

In contrast to expression differences, sequence divergence among alleles was often very high (Fig. 3; Fig. 4). Silent (synonymous and noncoding) divergence (d_S_) was over 5% in at least one pairwise comparison for 12 genes, and over 10% for 4 genes. All coding genes harbored nonsynonymous variants, but for non-TM1 coding genes, pairwise nonsynonymous divergence (d_N_) was 1% or less. In contrast, six of the TM1 genes had mean d_N_ >5%, and all of these had at least one 75bp window with d_N_ >15% in at least one comparison. The alleles at the remaining TM1 gene, *grctm1*, showed d_N_ <1% but still encoded very different proteins because the S2 allele had a premature stop codon approximately 30% of the way through its open reading frame. Because d_S_ was also very high among TM1 alleles, d_N_ did not significantly exceed d_S_. Only two genes showed high nonsynonymous substitution (>3%) specific to the R allele: *grctm5* (both R isoforms) and *grctm6*. Thus, the TM1 genes not only show structural similarity to known immune-relevant receptor genes, they also show the highest d_N_ among alleles of all the genes in the GRC.

### The GRC appears to be under balancing selection

In this snail population the GRC harbors three highly divergent haplotypes that have very even allele frequencies (>25%). In order to test for the signature of balancing selection, we compared Tajima's D and nucleotide diversity (π) for the GRC to that in the rest of the genome. Tajima’s D at Sanger-sequenced marker locus *grc1* (15 polymorphisms, 289 samples) is 3.99. Tajima’s D in the GRC cannot be directly estimated from the RNA-Seq data because samples were chosen non-randomly based on GRC genotype. However, if we adjust RNA-Seq SNP frequencies based on the population allele frequencies of the three haplotypes, Tajima’s D in the GRC is estimated as 3.85. In order to compare this estimate to genome-wide patterns, we calculated Tajima’s D in two independent datasets: the RNA-Seq data from the 36 unchallenged snails, and the RAD data from the 28 GUA snails. We identified 779 genomic scaffolds for which we could confidently align at least 15 RNA-Seq SNPs with no missing genotypes (15–157 SNPs per scaffold; excludes SNPs in the GRC). Genome-wide, mean Tajima’s D is significantly less than in the GRC (mean = 1.68; 95% CI = -0.98 to 3.62; empirical p for D ≥ 3.85 is 0.01). In the RAD data, only 709 variants had no missing data across all GUA samples, and only a single scaffold contained at least 15 such variants, so we could not estimate a distribution of Tajima’s D as we did with the RNA-Seq data. However, overall Tajima’s D for these RAD variants is 1.76, a very similar estimate to the RNA-Seq data, and again much lower than at the GRC. Both genome-wide estimates are still significantly higher than the neutral expectation of D = 0 (coalescent simulation, p < 0.05).

Similarly, silent and noncoding diversity (π_S_) in the GRC ranged among genes from 0.16% to 7.40%, with a majority of genes (15 out of 26 universally expressed genes) showing π_S_ > 2%. Nonsynonymous diversity (π_N_) in the GRC ranged among genes from 0.07% to 5.69%, with six TM1 genes showing π_N_ > 3%. In order to compare π in the GRC to genome-wide patterns, we identified 3061 genomic scaffolds for which we could confidently align at least 100 contiguous bp of RNA-Seq reads at high coverage (length = 100–11,694bp; excludes reads in the GRC). Genome-wide π is significantly lower than for most GRC genes (mean = 0.49%; 95% CI = 0.06–1.75%; empirical p for π ≥ 2% is 0.01; empirical p for π ≥ 3% is 0.001).

### BAC sequencing refines the assembly of the GRC

Scaffold1 includes the most interesting candidate genes as well as some obvious assembly errors (e.g. Scaffold7477 must be inserted somewhere). Therefore, in order to verify the assembly of the Scaffold1 portion of the GRC, we sequenced eight BAC clones that putatively tiled across the GRC region, and aligned them to the *B*. *glabrata* reference genome (BAC clones and the genome assembly were created using a Brazilian strain, BB02; [37]). All aligned to Scaffold1 as expected and largely confirmed the reference genome assembly between sites 1.471–1.971Mb, with a few exceptions. One large exception is the insertion (relative to the genome assembly) of a 69kb region aligning to Scaffold4587, Scaffold7002, and Scaffold7477. This complete insertion is found in two BACs, which also overlap on Scaffold1 between sites 1.74–1.80 Mb. This result is consistent with our RNA-Seq transcripts, one of which (*plmt*) is on Scaffold7477 but in perfect LD with the GRC, and one of which (*grctm2*) is partially on Scaffold7477 and partially on Scaffold1, sites 1.79–1.81 Mb. This insertion is accompanied by a 14kb deletion between sites 1.762–1.776 Mb on Scaffold1. Notably, this deletion contains a putative gene encoding an acetylglucosaminyltransferase. Thus this gene is not part of the GRC, consistent with RNA-Seq results. A second insertion of indefinite size (at least 67kb) occurs after Scaffold1 site 1.971Mb, as observed in two BACs. We did not observe sequence corresponding to the end of Scaffold1 from site 1.971Mb to 2.184Mb, which may occur after this final insertion or may have been incorrectly assembled onto Scaffold1. A small but interesting insertion matching Scaffold866, sites 57.7–58.1kb, is an exon of gene *grctm6*, corresponding exactly to the region of the gene where allele S1 shows no sequence similarity to the other alleles (Fig. 4).

## Discussion

We have identified and characterized a region of the *B*. *glabrata* genome, the GRC, that greatly influences immunity to schistosome parasites. Three major lines of evidence demonstrate the functional importance of the GRC. First, this region responded rapidly to selection for resistance in two independent experiments (Fig. 1A, Fig. 2). Second, it shows a strong and significant association with infection status in the unselected population of snails (Fig. 1B), explaining 23% of the variance in resistance in the population. Third, genetic diversity at this locus shows an unusual pattern with three highly divergent alleles in nearly perfect LD over hundreds of kilobases, suggestive of balancing selection (Fig. 3, Fig. 4).

### Genes in the GRC

The GRC contains 15 coding genes, some of which are duplicated within some haplotypes. Pinpointing the specific causal gene(s) responsible for resistance in this system will require additional experimental evidence, but the most promising candidates are the TM1 genes, especially those with many nonsynonymous variants unique to the resistant haplotype. Gene regulation in cis is not a strong candidate for the functional mechanism because there were few significant and/or large (>2-fold) expression differences, especially in coding genes (S1 Fig; S2 Fig). Although we cannot rule out a role for subtle expression differences, including in noncoding transcripts, the simplest mechanism of dominant resistance via gene expression would be overexpression of the resistance allele in a coding gene, and such a pattern is never observed. In contrast, the strikingly high amino acid sequence divergence is a much stronger candidate for the functional mechanism. All genes show some nonsynonymous differences, but only the TM1 genes show high d_N_ (Fig. 3). The TM1 alleles also harbor many length polymorphisms, and some alleles with short open reading frames may be nonfunctional (e.g. *grctm1*_S2, *grctm5*_S1, *grctm7*_S1, Fig. 4). We also note that the cluster of TM1 genes is in the center of the region of statistical association with resistance, the most likely position in which to find the causal locus or loci.

That these TM1 genes also code for proteins that share structural similarity to known pathogen-recognition molecules [36] is particularly intriguing. Typical TM1s usually have their transmembrane domain very close to the N- or C-terminal (<50 residues) [38], but in all seven GRC TM1 genes, the transmembrane domain is >50 residues from either end, a feature they share with Toll-like receptors and a minority of other TM1s. Most also contain fibronectin III domains which are often involved in molecular recognition [39]. Thus, although not all the TM1 genes show sequence similarity to each other, they may be functionally or even evolutionarily related. Specifically, we hypothesize that their extracellular (N-terminal) domains recognize foreign substances such as parasite PAMPs (pathogen-associated molecular patterns), while their intracellular (C-terminal) domains transmit this signal to other cellular components, leading to a physiological response. For example, *S*. *mansoni* produces polymorphic mucins, SmPoMucs [40], that interact with snail FREP immunity proteins [20,41,42]. It is possible that the TM1 proteins recognize SmPoMucs, SmPoMucs-FREP complexes, or unrelated PAMPs.

The most compelling candidates for controlling resistance to *S*. *mansoni* in the TM1 gene cluster are *grctm5* and *grctm6*. Only these two genes show high nonsynonymous substitution specific to the R haplotype (Fig. 4). Gene *grctm5* encodes a fibronectin III protein with sequence similarity to chitinase. Intriguingly, it is present in two copies in RR snails but only one copy in the susceptible genotypes. Furthermore, these two R isoforms are substantially divergent from each other (d_N_ = 7.9%, d_S_ = 7.4%), as well as from S1 (d_N_ = 4.2–6.8%, d_S_ = 7.1–7.8%) and S2 (d_N_ = 7.4–7.5%, d_S_ = 6.7–9.1%) alleles. Both *grctm5* R sequences show large, significant expression differences between RR (depth = 2.8x, 3.0x) and S1S1 (depth = 1.0x; t-tests, p < 10^-3^ for both), but not between RR and S2S2 (2.3x; t-tests, p >0.1 for both) (S1 Fig). Gene *grctm6* has no strong sequence similarity to any known proteins. It shows high divergence among all three haplotypes, and includes a ~300bp segment where the S1 haplotype shows no sequence similarity to R or S2. The *grctm6* gene also shows significantly lower expression in RR than in S1S1 or S2S2.

### Balancing selection on the GRC and host-parasite coevolution

The GRC, especially the TM1 genes, shows three patterns of genetic variation consistent with balancing selection. First, with three intermediate frequency alleles, the site-frequency spectrum is highly skewed away from neutral expectation, as evidenced by the extremely high Tajima's D value. Second, high LD across several Mb is an unusual pattern suggestive of selection [43]. Although high LD can also be caused by low recombination rates, this explanation seems unlikely. In most genomes, regions of low recombination are gene-poor [44], unlike the GRC. The main exceptions are chromosomal inversions which can suppress recombination across suites of genes; however, an inversion cannot maintain high divergence among more than two haplotypes, so the three divergent haplotypes of the GRC require another explanation. Both Tajima’s D and LD can be elevated by demographic bottlenecks. Genome-wide values of Tajima’s D in the Guadeloupe laboratory population are higher than the neutral equilibrium expectation, suggesting such a demographic effect. However, Tajima’s D at the GRC is significantly higher still, suggesting that selection has also played a role. Because the Guadeloupe laboratory population has had a census size in the hundreds at collection and ever since, we expect genetic drift in these snails to have been minimal. Thus, both allele frequencies and the extent of LD at the GRC should be similar to values in the wild, which we cannot directly estimate. Guadeloupe *B*. *glabrata* undergoes population fluctuations in the wild [45], and these natural demographic dynamics likely contribute to the genome-wide population genetic patterns. In addition, our genome-wide estimates of Tajima’s D are based on samples that did experience minor laboratory bottlenecks (N = 100 over five generations for GUA RAD; N = 30 for the founding of the Oregon State University population from which the RNA-Seq data is derived). Therefore, genome-wide Tajima’s D and LD may be lower in the wild, and the GRC may be an even more extreme outlier.

The third non-neutral pattern, high genetic diversity, cannot be explained by demographics. The GRC is an outlier with respect to nucleotide diversity (π) at both synonymous and nonsynonymous sites, especially in six of the TM1 genes. The high nonsynonymous diversity (π_N_ = 3.2–5.7%) observed at six of the TM1 genes is quite remarkable, as it greatly exceeds the presumably neutral values seen throughout most of the genome (p < 0.001). Of course, reads at highly diverse loci might be less likely to align to the reference genome, so our genome-wide estimate of π is likely biased downward. Still, such high nonsynonymous diversity is rarely seen in any species; for example, 99.9% of *Drosophila simulans* genes show lower π_N_ values [46]. We did not observe a d_N_/d_S_ ratio significantly greater than one at any gene. At *grctm6*, d_N_ is 1.65 times as high as d_S_ (synonymous plus noncoding) divergence (Fig. 3), but only 0.83 times the magnitude of synonymous divergence alone (excluding noncoding sites). Such high diversity at both synonymous and nonsynonymous sites is evidence that alleles have coexisted for an unusually long time at intermediate frequency, presumably because selection has maintained them in balance [47,48]. As with other immunity genes that show similar patterns [49], it may be the case that heterozygotes have a fitness advantage because they can recognize and respond to a greater diversity of parasites, or it may be that parasite community composition changes so quickly that no one allele is advantageous for long enough to fix. Because *S*. *mansoni* arrived in the New World only in historical times [50], the selection pressure maintaining these alleles must be due to other native parasites, perhaps including other trematodes which have a very close and ancient relationship with snails [17,51]. As with Tajima’s D and LD, π in the Guadeloupe laboratory population may be slightly different than in the wild, although we expect the effect of genetic drift in the lab to have been minimal. If anything, we would expect even greater diversity in natural populations, as there may be additional unsampled divergent alleles, especially if populations outside of Guadeloupe are considered (indeed, the reference genome provides an example). Additional work is needed to study the relevance of the GRC to phenotypic variation in resistance across the range of *B*. *glabrata*, or in other snail species.

Théron et al. [33] showed that the shape of dose-response curves in the Guadeloupe snail-schistosome community is consistent with a simple host-parasite phenotype compatibility model, in which allelic variation in the snails controls matching with the parasite, likely via a complimentary locus (or loci) in the Guadeloupe population of *S*. *mansoni*. Such phenotype compatibility could occur by several mechanisms. Under the matching alleles (MA) model, parasites avoid detection by matching host self determinants, whereas under the inverse matching allele (IMA) model, host molecules recognize parasite molecules leading to an immune response [34]. Importantly, heterozygous hosts are susceptible under MA and resistant under IMA [34]. Thus, the genetic dominance of resistance at the GRC favors IMA.

Similar signatures of balancing selection are seen across a wide range of host taxa at immunity loci that interact directly with infectious disease agents [52–54]. In the *Anopheles*/*Plasmodium* pair, the best studied system of invertebrate host and eukaryotic parasite, several genomic loci have been associated with host resistance and show patterns paralleling those seen at the GRC [55–57]. For example, the *Anopheles APL1* locus also represents a cluster of structurally similar genes that show extraordinarily high nonsynonymous diversity of the same magnitude as the GRC TM1 genes (3–6%) [55,57]. Likewise, nonsynonymous divergence is high among alleles of the *Anopheles TEP1* locus, although gene conversion rather than balancing selection appears to be responsible [56]. Nonsynonymous diversity exceeding neutral expectations is also consistently observed in the MHC of vertebrates [49] and plant R genes [58]. As with the GRC, genetic dominance of resistance is common across these systems, consistent with the IMA model. Furthermore, the close physical linkage of the TM1 genes resembles the gene clusters that form many of these resistance genomic regions in other taxa. Such clustering may be a neutral artifact of tandem gene duplication, or it may have adaptive significance through shared gene regulation or the maintenance of beneficial multi-gene haplotypes [59].

### Possible applications

The GRC is an obvious target for applications in the control of schistosomiasis. Genetic manipulation of disease vectors is a promising approach that is already underway for mosquitoes and other pest species [9,60,61], and driving resistance genes into *B*. *glabrata* populations is under discussion [27]. Gene knockdowns in *B*. *glabrata* have shown repeated success [23,24,62,63], and targeted inhibition of GRC genes or their products may pinpoint the specific causal gene. Alternatively, transfection with a multi-gene haplotype could have practical utility even without perfect understanding of each gene’s functional contribution. Although genetic modification of snails is still in its infancy, the CRISPR nuclease system shows promise for fine-scale modification of even non-model species [9]. Importantly, transgenic vectors would not need to have higher fitness than wild-type organisms if transgenes were spread by gene drives [9]. Thus, if only the R allele recognizes *S*. *mansoni* while other alleles recognize other parasites of no importance to human health, it would be possible to drive the R allele to fixation even without an adaptive benefit to the snails, if it were coupled to a gene drive conferring preferential non-Mendelian inheritance of the R allele [9]. Conversely, if multiple alleles at this locus are required to recognize all strains of *S*. *mansoni*, it might be possible to engineer a single haplotype with duplicated genes that included all relevant sequences. Alternatively, if the GRC genes initiate an immune signal cascade upon recognition of the parasite, future research could seek to manipulate snails such that this signal cascade is constitutively upregulated regardless of genotype or infection status. The matching loci in the parasite, once they are identified, may also be targets for drugs or genetic manipulation.

## Materials and Methods

### Ethics statement

Mice and hamsters were used to maintain the schistosome parasites and to produce miracidia for challenge experiments. Infection is through contact with inoculated water and involves minimal discomfort. Infected rodents are euthanized with CO_2_ prior to showing clinical signs of disease and are dissected to recover parasitic worms and their eggs. This research was approved by OSU IACUC and the French veterinary agency.

### Experimental evolution

*B*. *glabrata* and *S*. *mansoni* were collected on the island of Guadeloupe, West Indies, in 2005 as described previously [33]. Selection lines were created at the University of Perpignan beginning in 2008. We selected for resistance by challenging snails with 10 (line R10) or 30 (line R30) *S*. *mansoni* miracidia and allowing only uninfected snails in each independent line to contribute to the next generation (N = 50–100 snails challenged per line per generation, and so the numbers used to found each subsequent generation varied each generation and increased from as low as 38 to 90 as the susceptibility of the two lines decreased; S1 Table). This was done for five generations. We measured susceptibility in each line as the percentage of snails infected (Fig. 1A). As a control, we maintained a line of uninfected snails (GUA) at a similar population size for the same time period (N = 100 snails per generation). For GUA, 50–70 snails from each offspring generation were challenged in order to measure the susceptibility of GUA each generation (percentage of snails infected, S1 Table), but these challenged snails did not contribute to subsequent generations. We tested for a correlation between generation and susceptibility by logit transforming susceptibility and using linear regression. We estimated the standard errors of proportions using √((p*(1-p))/N).

### RAD genotyping

We used *SbfI* in RAD genotyping [64] of 28 individuals each from the control population (GUA) and the two selection lines (R10 and R30) (Illumina data at NCBI SRA, Bioproject Accession PRJNA268191). We aligned reads to the *B*. *glabrata* reference genome with BWA [65]. All genomic analyses in this study used version 4.3 of the reference genome, and positions were subsequently converted to nearly identical reference genome version BglaB1 (https://www.vectorbase.org/organisms/biomphalaria-glabrata). In order to maximize the density of markers, we considered both markers with observable variants as well as null markers for which a RAD tag aligned in only a subset of individuals. To estimate allele frequencies of SNPs, we counted two alleles per individual with 10x or greater depth, and two half-alleles per individual with 2–9x depth to account for possible nonobserved alleles, while genotypes with <2x depth were counted as missing. We only analyzed sites with a per-population allele count of at least 24 for both GUA and at least one other population, and which did not violate Hardy-Weinberg equilibrium (χ^2^ > 20) in any population nor show overall heterozygote excess among populations. Allele frequencies for null markers were estimated using the method of Zhivotovsky [66]. For each informative marker we estimated the difference in allele frequency between GUA and each selected population (F_ST_) as 1 –(mean within-population expected heterozygosity)/(total expected heterozygosity). To assess RAD tags with low sequence similarity to the reference genome, we aligned RAD tags to each other with Stacks [35] and tested for any previously undetected high-F_ST_ markers. We assessed the robustness of F_ST_ results with 100 bootstrap replicates in which 28 samples from each population were chosen randomly with replacement. We chose the single most extreme outlier (highest F_ST_), along with markers showing evidence of close physical linkage to this marker (<1Mb away on same scaffold and/or high LD), for further analysis. For these promising markers, we designed primer pairs to amplify and Sanger sequence the RAD tag in order to obtain the complete codominant genotype for all individuals.

### Sanger verification

We phenotyped an independent sample of 289 snails by challenging them with 20 miracidia each and then designating them as either infected or uninfected following Theron et al. [33]. We examined markers showing exceptionally high F_ST_ by Sanger sequencing them in these 289 snails and testing for an association with phenotype. We calculated allele frequencies and standard errors of proportions. We calculated the proportion of phenotypic variation explained by the R allele as the square of the correlation coefficient (r^2^) from a logistic regression of phenotype (infected or not) versus genotype (R/- or not). We then developed primers to amplify nearby genomic regions in order to find and characterize the genomic region showing the strongest association with resistance. We tested these markers on a subset of 94 phenotyped snails.

### RNA-Seq of unchallenged snails

We extracted RNA from whole bodies of randomly-chosen, size-matched juvenile snails that had not been parasite-challenged. We prepared samples using the TruSeqTM RNA v2 kit (Illumina RS-122–2001) following the low-throughput protocol found in the Sample Preparation v2 Guide. We performed RNA-Seq on the following 36 samples: 12 RR homozygotes (single-end), 6 RR homozygotes (paired-end), 6 S1S1 homozygotes (single-end), 3 S1S1 homozygotes (paired-end), 6 S2S2 homozygotes (single-end), and 3 S2S2 homozygotes (paired-end) (we first Sanger genotyped a large number of randomly-chosen snails from tentacle snips, and then we chose the above 36 to use for RNA-Seq). Sequencing was conducted on the Illumina HiSeq 2000 at Oregon State University (Illumina data at NCBI SRA, Bioproject Accession PRJNA264063). We converted FASTQ files to FASTA and used Jellyfish 1.0.2 [67] to count 31-mers in each sample, which is the largest kmer size that Jellyfish can count, but which is long enough to be typically unique in the genome and therefore represent specific transcripts. We then used a custom perlscript (https://github.com/jacobtennessen/HOLDRS) to identify 31-mers that differed significantly among genotypes, defined as showing at least a 2-fold difference in total count and a Welch’s t-statistic of at least 5. These parameters were chosen to encompass both sequence and expression differences: expression differences under 2-fold may not be biologically meaningful, true sequence differences should result in a much larger (all or nothing) count ratio, and this t-statistic typically corresponds to a p-value under 10^-4^, which will minimize the number of false positives among 31-mers from thousands of transcripts, while still detecting 31-mers that are truly overabundant in one sample (N = 9 or 18) versus a control (N = 18). We identified reads containing these divergent 31-mers and used ABySS v. 1.3.4 [68] to assemble them into contigs. We used grep and manual alignment to extend these contigs and assemble additional contigs from divergent reads. We focused subsequent analysis on contigs >500bp, as well as shorter contigs with putative orthology to a contig >500bp in another genotype (sequences in NCBI Transcriptome Shotgun Assembly Database, Bioproject Accession PRJNA264063). In order to measure sequencing depth and identify polymorphisms, we aligned reads to these long contigs using BWA [65] and converted genotypes to vcf format.

### RNA-Seq of challenged snails

We chose six snail lineages homozygous at the GRC (3 RR, 1 S1S1, 2 S2S2). For each family we exposed 6 randomly-chosen, size-matched juvenile individuals to 20 miracidia each, and then we extracted RNA from whole bodies at 2 hr (n = 3) and 6 hr (n = 3) post infection. RNA from each set of 3 snails from the same family and time point was then pooled. We prepared and sequenced samples single-end as described above (Illumina data at NCBI SRA, Bioproject Accession PRJNA264063). We aligned reads to the assembled transcripts from the unchallenged dataset and compared depths as described above.

### BAC sequencing

We BLASTed the GRC portion of Scaffold1 (*B*. *glabrata* reference genome) against end sequences from the BAC library generated for *B*. *glabrata* strain BB02 (ftp://ftp.ncbi.nih.gov/pub/TraceDB/biomphalaria_glabrata/; [37]). We chose BACs from among the hits, ordered them from the Arizona Genomics Institute, extracted DNA, and sequenced them in a Nano run of the Illumina MiSeq at Oregon State University (Illumina data at NCBI SRA Bioproject Accession PRJNA268208). We aligned the reads from eight BACs to the *B*. *glabrata* reference genome with BWA [65].

### Analysis

We calculated linkage disequilibrium between markers as the correlation coefficient, r, in number of R alleles per genotype (0, 1, or 2) We classified transcripts as “coding” if they contained an open reading frame of at least 500bp, otherwise they were designated “noncoding.” We used DELTA-BLAST [69] to match candidate transcripts with known protein families. Genes were named following the guidelines in Bayne [70]. We characterized secondary structure with Jpred3 [71] and identified transmembrane domains using TMHMM v. 2.0 (http://www.cbs.dtu.dk/services/TMHMM/). We measured pairwise synonymous (d_S_) and nonsynonymous (d_N_) divergence, as well as allele-specific nonsynonymous substitution, with custom Perl scripts and DnaSP [72]. For each transcript, we used t-tests to identify significant differences in adjusted per-sample, per-site depth among genotypes in which all values were divided by the ratio of the total count of reads in that individual relative to the mean per-individual read count, and then log-transformed. Depth values of zero were arbitrarily recorded as 1/6, which assumes they are present but too rare (much less than 1x) to have been sampled. For each transcript under each experimental scenario (unchallenged, 2 hours post-challenge, and 6-hours post-challenge) we compared RR vs. SS. For unchallenged snails we also compared RR vs. S1S1 and RR vs. S2S2. If multiple sequences of the same gene were observed for one genotype, these were all tested separately. In order to estimate Tajima’s D [73] in the GRC, we calculated the frequency of all RNA-Seq SNPs on their haplotype (R, S1, or S2), and then multiplied this frequency by the unbiased haplotype frequency estimate from the 289 snails Sanger sequenced at *grc1*. In order to estimate genome-wide Tajima’s D and π, we aligned RNA-Seq reads to the *B*. *glabrata* reference genome with BWA [65]. We identified regions with at least 100 contiguous bp showing high (≥6x) depth in all 36 samples. All of these high-depth regions were used to estimate π, while scaffolds with at least 15 SNPs in such high-depth regions were included in the estimate of Tajima’s D. Significance of Tajima’s D was estimated with standard neutral coalescent simulations in DnaSP [72].

## Supporting Information

S1 Fig Expression of genes comprising the Guadeloupe Resistance Complex (GRC) in parasite-unchallenged snails. As in Fig. 3, genes are aligned to their approximate genomic position on the x-axis, and Scaffolds 1, 4, and 7477 have been oriented based on their estimated genomic positions. Expression (per-sample, per site depth) in all three homozygous genotypes (indicated by color) is shown on the left y-axis (log scale), with standard error bars. Gene symbols vary for ease of distinguishing adjacent genes, and to show gene type: open symbols (circles and triangles) represent coding genes (>500 bp open reading frame), while other symbols represent noncoding genes. When more than one sequence was observed for the same gene from a particular haplotype, all are plotted separately. Only two genes, both non-coding, show a >2-fold, significant difference between RR and SS genotypes: *grcnc8* and *grcnc9*. Brown lines and squares indicate the boundaries of the GRC as in Fig. 3, with linkage disequilibrium (r; Table 1) to *grc1* (labeled) indicated on the right y-axis.
(PDF)

S2 Fig Expression of genes comprising the Guadeloupe Resistance Complex (GRC) in parasite-challenged snails. As in Fig. 3, genes are aligned to their approximate genomic position on the x-axis, and Scaffolds 1, 4, and 7477 have been oriented based on their estimated genomic positions. Expression (per-sample, per site depth) for genotypes (indicated by color) is shown on the left y-axis (log scale), with standard error bars (data from S1S1 and S2S2 families merged due to small sample size). Gene symbols vary for ease of distinguishing adjacent genes, and to show gene type: open symbols (circles and triangles) represent coding genes (>500 bp open reading frame), while other symbols represent noncoding genes. When more than one sequence was observed for the same gene from a particular haplotype, all are plotted separately. Only four genes show a >2-fold, significant difference between RR and SS genotypes for at least one time interval: *grcnc2*, *grctm6*, *hely*, and *grcnc8*. There were no coding genes with increased expression in RR families. Brown lines and squares indicate the boundaries of the GRC as in Fig. 3, with linkage disequilibrium (r; Table 1) to *grc1* (labeled) indicated on the right y-axis. (A) Expression 2 hours post-challenge. (B) Expression 6 hours post-challenge.
(PDF)

S1 Table Selection for parasite resistance. For three experimental populations (R10, R30, and GUA), snail susceptibility phenotypes were measured over five generations.
(XLS)
