# Supplementary figures and images for: Hyperdiverse Gene Cluster in Snail Host Conveys Resistance to Human Schistosome Parasites
Source: PLoS Genet. 2015 Mar 16;11(3):e1005067. doi: 10.1371/journal.pgen.1005067 (PMC4361660; doi:10.1371/journal.pgen.1005067)

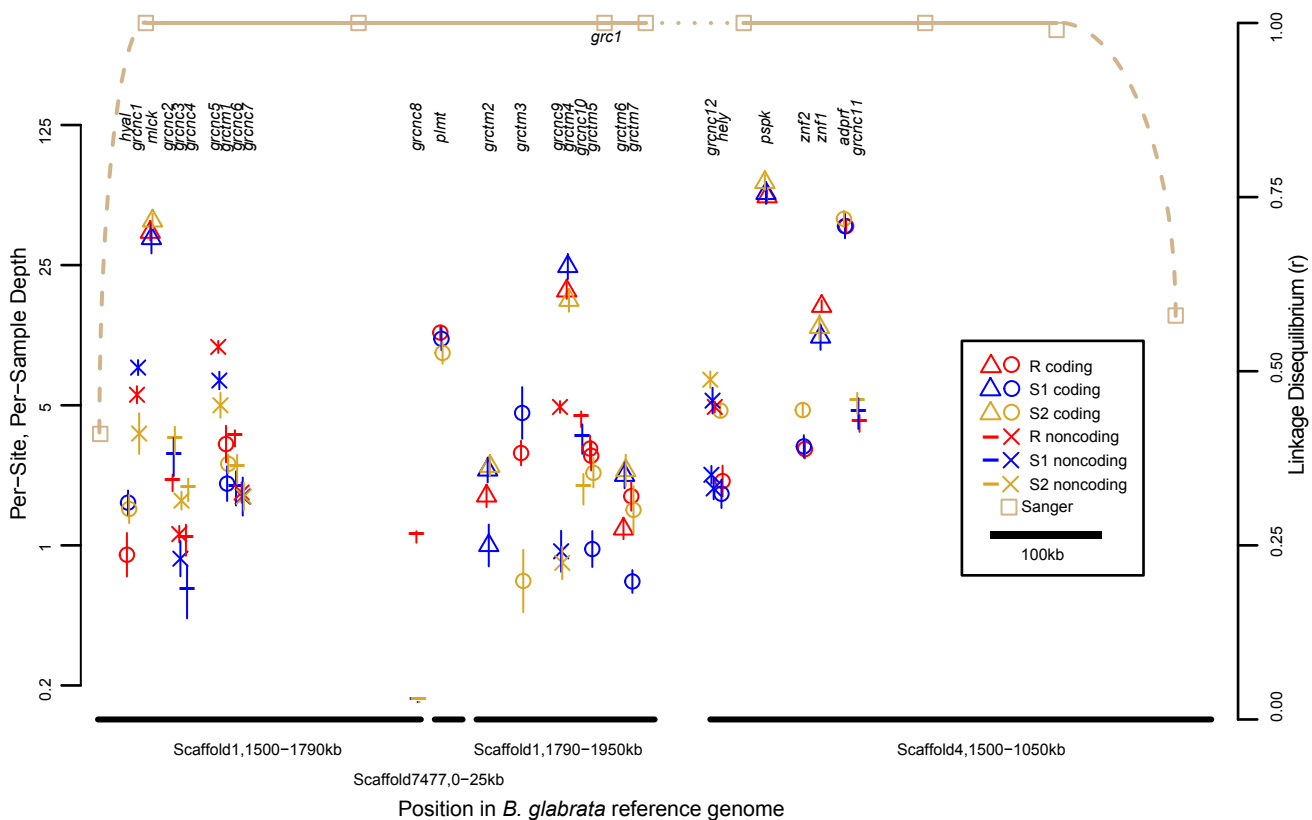

Supplement: S1 Fig — As in Fig. 3, genes are aligned to their approximate genomic position on the x-axis, and Scaffolds 1, 4, and 7477 have been oriented based on their estimated genomic positions. Expression (per-sample, per site depth) in all three homozygous genotypes (indicated by color) is shown on the left y-axis (log scale), with standard error bars. Gene symbols vary for ease of distinguishing adjacent genes, and to show gene type: open symbols (circles and triangles) represent coding genes (>500 bp open reading frame), while other symbols represent noncoding genes. When more than one sequence was observed for the same gene from a particular haplotype, all are plotted separately. Only two genes, both non-coding, show a >2-fold, significant difference between RR and SS genotypes: grcnc8 and grcnc9. Brown lines and squares indicate the boundaries of the GRC as in Fig. 3, with linkage disequilibrium (r; Table 1) to grc1 (labeled) indicated on the right y-axis. (PDF) [file pgen.1005067.s001.pdf]

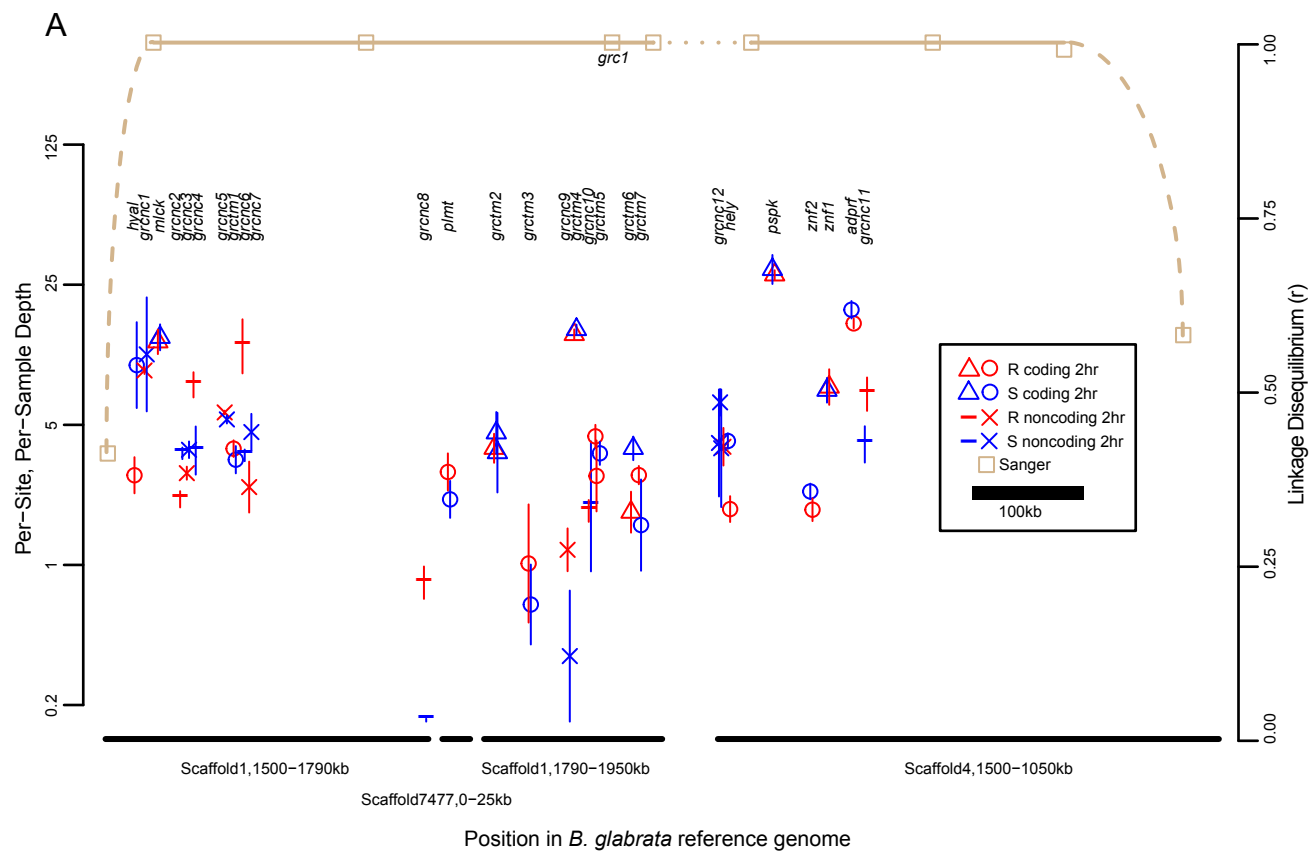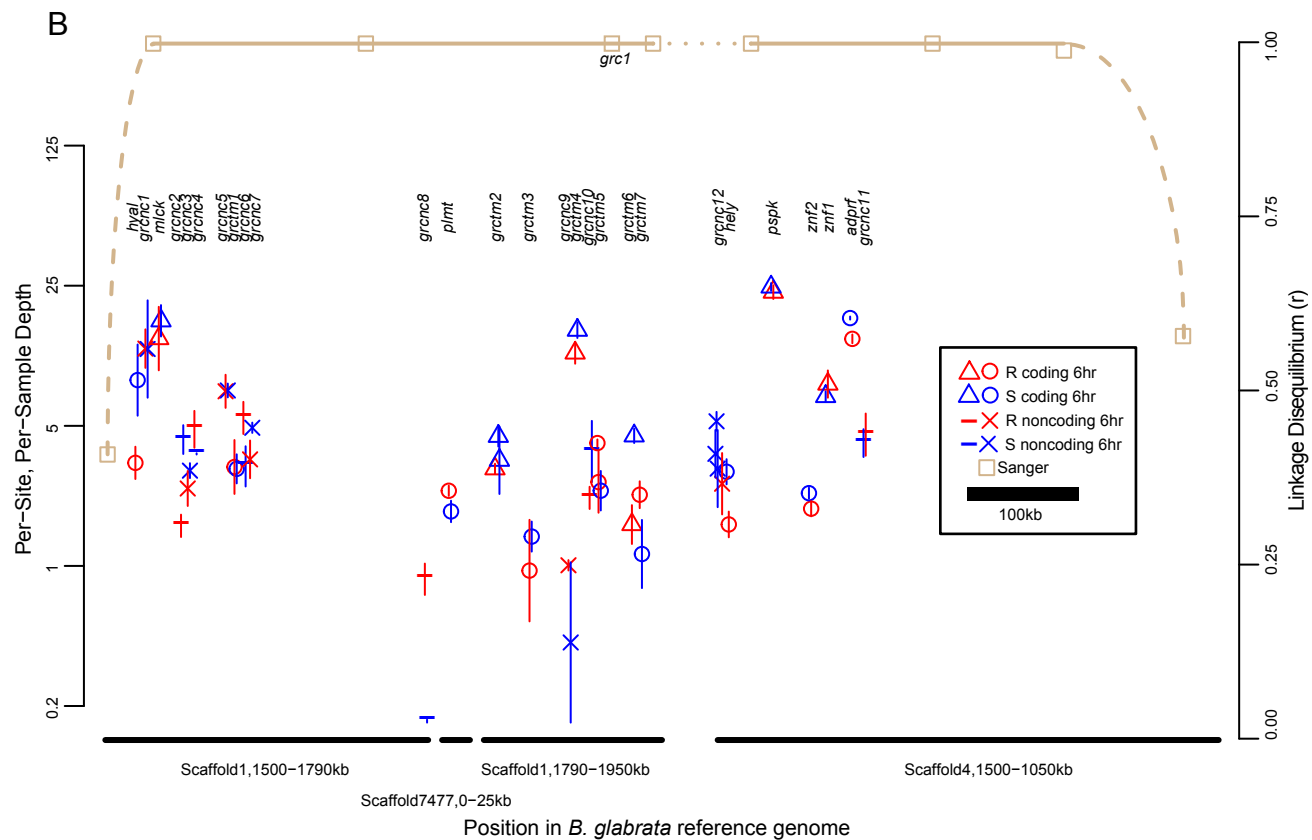

Supplement: S2 Fig — As in Fig. 3, genes are aligned to their approximate genomic position on the x-axis, and Scaffolds 1, 4, and 7477 have been oriented based on their estimated genomic positions. Expression (per-sample, per site depth) for genotypes (indicated by color) is shown on the left y-axis (log scale), with standard error bars (data from S1S1 and S2S2 families merged due to small sample size). Gene symbols vary for ease of distinguishing adjacent genes, and to show gene type: open symbols (circles and triangles) represent coding genes (>500 bp open reading frame), while other symbols represent noncoding genes. When more than one sequence was observed for the same gene from a particular haplotype, all are plotted separately. Only four genes show a >2-fold, significant difference between RR and SS genotypes for at least one time interval: grcnc2, grctm6, hely, and grcnc8. There were no coding genes with increased expression in RR families. Brown lines and squares indicate the boundaries of the GRC as in Fig. 3, with linkage disequilibrium (r; Table 1) to grc1 (labeled) indicated on the right y-axis. (A) Expression 2 hours post-challenge. (B) Expression 6 hours post-challenge. (PDF) [file pgen.1005067.s002.pdf]
